# Combination therapy targeting toll like receptors 7, 8 and 9 eliminates large established tumors

**DOI:** 10.1186/2051-1426-2-12

**Published:** 2014-05-13

**Authors:** By Gan Zhao, John P Vasilakos, Debra Tross, Dmitri Smirnov, Dennis M Klinman

**Affiliations:** 1 Cancer and Inflammation Program, National Cancer Institute, NIH, Frederick MD 21702, USA; 2 3M Drug Delivery Systems Division, St. Paul MN 55144, USA

**Keywords:** Cancer, Therapy, TLR agonists, CpG ODN, Innate immunity

## Abstract

**Background:**

The TLR7/8 agonist 3M-052 and the TLR9 agonist CpG ODN both trigger innate immune responses that support the induction of tumor-specific immunity. Previous studies showed that these agonists used individually could improve the survival of mice challenged with small tumors but were of limited therapeutic benefit against large/advanced tumors.

**Methods:**

Normal mice were challenged with syngeneic tumors. Once these tumors reached clinically detectable size (500–800 mm^3^) they were treated by intra-tumoral injection with 3M-052 and/or CpG ODN. Anti-tumor immunity and tumor growth were evaluated.

**Results:**

The co-delivery of agonists targeting TLRs 7, 8 and 9 increased the number and tumoricidal activity of tumor infiltrating CTL and NK cells while reducing the frequency of immunosuppressive MDSC. The combination of 3M-052 plus CpG ODN (but not each agent alone) eradicated large primary tumors and established long-term protective immunity.

**Conclusion:**

The combination of agonists targeting TLRs 7/8 and 9 represents a significant improvement in cancer immunotherapy.

## Background

Toll-like receptors (TLRs) comprise a family of highly conserved germline-encoded pattern recognition receptors that detect pathogen-associated molecular patterns (PAMPs) expressed by a variety of infectious microorganisms [1]. The ability of TLRs to trigger the innate immune system and bolster adaptive immunity against antigens expressed by pathogens and tumors is well established [2,3]. At least 13 different TLRs have been identified in mammals, with TLRs 7, 8, and 9 being similar in their recognition of nucleic acid motifs and expression within endosomal compartments [1,4,5].

Studies show that TLR7 is primarily expressed by plasmacytoid dendritic cells (pDC), TLR8 by monocytes, monocyte-derived (m)DCs, macrophages and Langerhans cells, and TLR9 by DCs, B cells, monocytes and mast cells [6-9]. Synthetic agonists designed to stimulate TLR7 typically trigger TLR8 as well and induce the secretion of IL-12 and TNFα by mDCs and/or pDCs [10,11]. Many TLR7/8 agonists also enhance the expression of co-stimulatory molecules and the migration of DCs, thereby facilitating the induction of Th1 immune responses [12,13]. Synthetic oligonucleotides that express CpG motifs trigger TLR9 and elicit a Th1-dominated immune response characterized by the production of pro-inflammatory cytokines (including IL-12, IFNα, and TNFα) and the up-regulation of co-stimulatory (CD80 and CD86) and MHC class I and II molecules [14-16].

The anti-tumor activity of TLR agonists targeting TLRs 7, 8 and 9 has generally been explored by delivering them systemically to mice with relatively small tumors (typically ≤200 mm^3^) [17]. While effective against tumors <300 mm^3^, TLR-based therapy of large tumors (>500 mm^3^) has been much harder to achieve [18-21]. A growing body of evidence suggests that the efficacy of TLR agonists might be improved by i) using them in combination and ii) injecting them directly into the cancerous tissue [18,22]. Large tumors are commonly infiltrated by immunosuppressive leukocytes that down-regulate anti-tumor responses. Local delivery of TLR agonists appears to interfere with the function of toleragenic cells in the tumor microenvironment. In this context, intra-tumoral injection of CpG ODN reduced the number and suppressive activity of tumor infiltrating MDSC [22]. Based on preliminary findings, we hypothesized that a combination of agonists targeting TLRs 7, 8 and 9 might be highly effective against established tumors. Unfortunately, the physicochemical characteristics of first generation TLR7/8 agonists resulted in a short *in vivo* half-life that reduced their activity when co-delivered with CpG ODN.

In the current work, this limitation was overcome by studying a novel TLR7/8 agonist (3M-052) modified with a lipophylic tail that persists *in vivo* at high levels for at least 24 hr after administration [23] (Additional file 1: Figure S1 and Additional file 2: Table S1). Results show that the combination of 3M-052 plus CpG ODN significantly increases CTL activity and Th1 cytokine production while down-regulating the activity of immunosuppressive MDSC. Although neither CpG ODN nor 3M-052 alone were effective against large tumors, the combination was highly active and mediated tumor eradication and the establishment of long-term immunity.

## Results

### Effect of TLR agonists on the growth of small tumors

**Figure 1:**
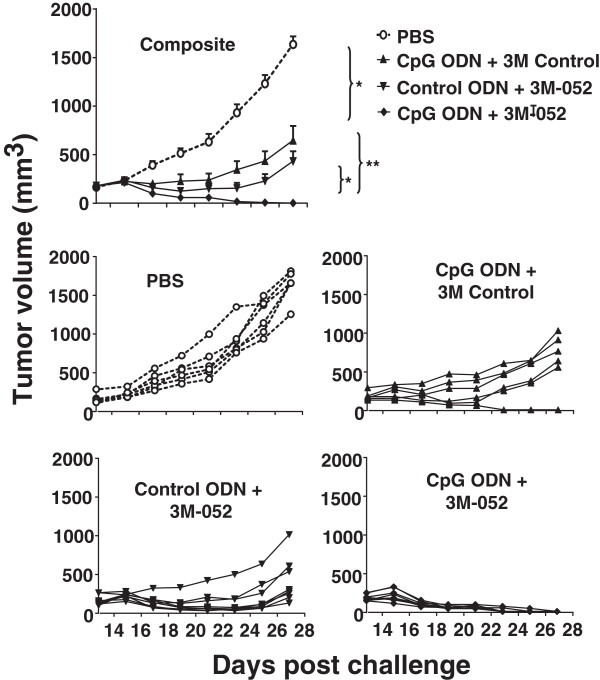
**Effect of TLR agonists on the growth of small tumors.** 10^5^ CT26 colon carcinoma cells were implanted into the flank of syngeneic BALB/c mice. When the tumors reached ≈ 200 mm^3^ in volume they were injected with 100 μg of CpG or control ODN and/or 50 μg of 3M-052 or 3M control. Each treatment was repeated 2 days later. Data show the change in tumor volume (mean + SE) of 6–8 mice from 2 independent experiments. *, p < 0.05; **, p <0.01 compared with the control group.

CT26 colon cancer cells were implanted subcutaneously into the flank of syngeneic BALB/c mice. When these tumors reached 200 mm^3^ in volume, 100 μg of ODN and/or 50 μg of 3M-052 was injected intra-tumorally and the procedure repeated 2 days later. Tumors in untreated mice proliferated rapidly and increased in size by 5-fold within 2 wk (Figure 1). The rate of proliferation was significantly reduced by treatment with either CpG ODN or 3M-052, although the tumors persisted. In contrast, animals treated with a combination of CpG ODN plus 3M-052 completely rejected their tumors (p < 0.01; Figure 1).

### Effect of TLR agonists on the frequency of tumor infiltrating mMDSC, NK and CD8 T cells

**Figure 2:**
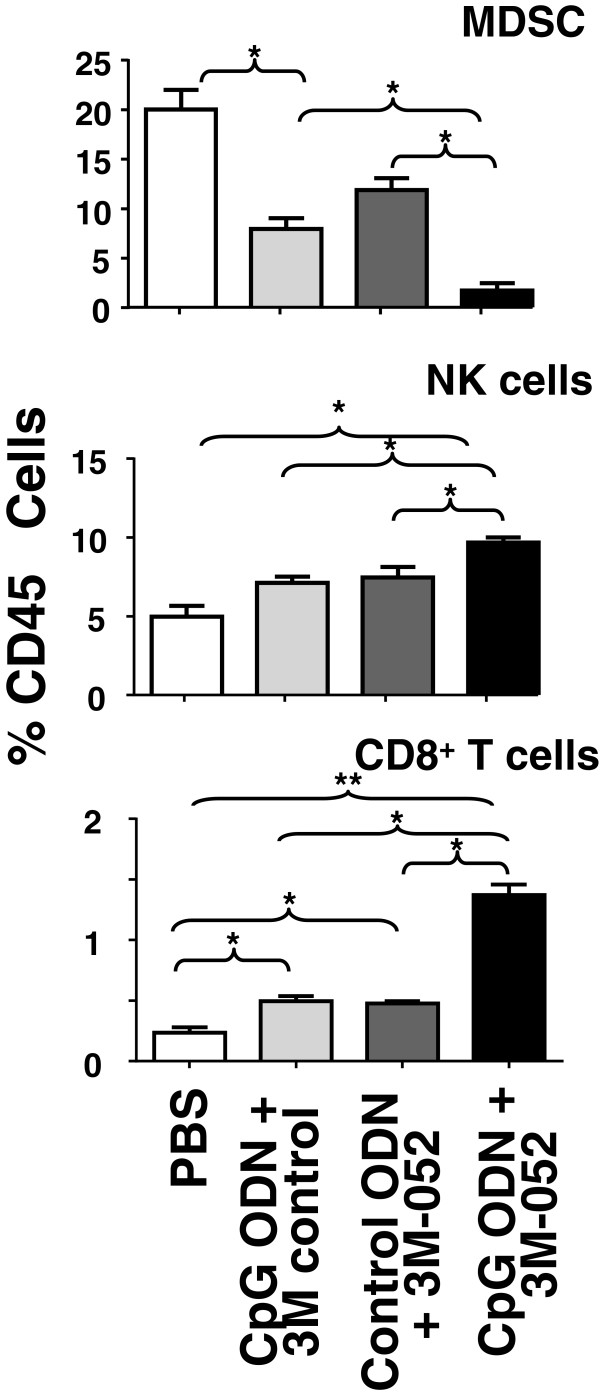
**Effect of TLR agonists on the frequency of tumor infiltrating MDSC, NK and CD8 T cells.** Mice were treated as described in Figure 1. The frequency of tumor-infiltrating MDSC, NK and CD8^+^ T cells was determined one day after the second treatment. Results show the mean + SD of each cell type as a percentage of total CD45^+^ tumor infiltrating cells analyzed independently in 6 mice from 2 independent experiments. *, p < 0.05; **, p <0.01.

Immune cells in the tumor microenvironment profoundly influence the success of immunotherapy. A single cell suspension was prepared from tumor samples, and the frequency of various immune subsets evaluated by FACS (Additional file 3: Figure S2). The number of mMDSC is considered an important marker of immune suppression, as these cells suppress the tumoricidal activity of CTL and NK cells. Consistent with previous reports, the frequency of Gr1^+^CD11b^+^ mMDSC was significantly elevated in mice bearing CT26 tumors (Figure 2). Treatment with either CpG ODN or 3M-052 alone reduced the number of mMDSC infiltrating the tumor site by ≈ 50% (p <0.05). The combination of these two agonists resulted in a nearly 90% reduction in mMDSC frequency (p <0.01, Figure 2). This effect was detectable by 1 day after the second treatment.

Previous studies showed that the infiltration of NK and CD8 T cells into the tumor microenvironment was associated with improved host survival [22]. The effect of TLR agonist treatment on the frequency of tumoricidal cells was therefore analyzed. The number of NK cells was ≈ 25% higher in mice treated with 3M-052 or CpG ODN when compared to untreated controls (p <0.05, Figure 2). This increase was magnified in mice treated with the combination of both TLR agonists. By comparison, while CpG ODN or 3M-052 alone increased CD8 T cell frequency by approximately 2-fold, the combination of both agonists synergistically increased CD8 T cell numbers by >5-fold (p <0.05, Figure 2). No effect on the frequency of Foxp3^+^ Treg was observed (Additional file 4: Figure S3).

**Figure 3:**
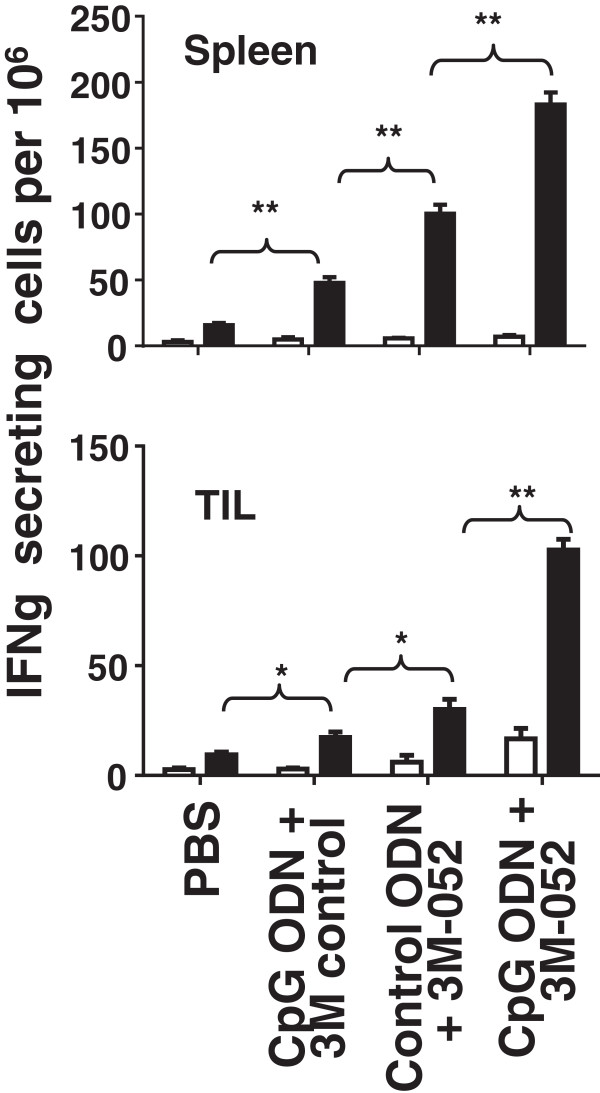
**Effect of TLR agonists on tumor-specific CTL.** CT26 tumors were implanted into BALB/c mice as described in Figure 1. Spleen cells and tumor infiltrating lymphocytes were isolated one day after the second treatment, stimulated *ex vivo* with AH-1 peptide, and monitored for IFNγ secretion by ELIspot assay. Results represent the mean + SD of 6 mice from 2 independent experiments. *, p < 0.05; **, p <0.01.

**Figure 4:**
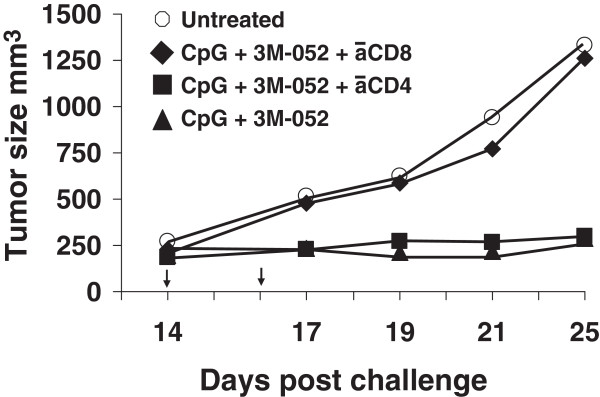
**Effect of depleting CD8 T cells on immune mediated protection.** CT26 tumors were implanted into BALB/c mice and treated with 100 μg of CpG and 50 μg of 3M as described in Figure 1. The effect of depleting CD4 or CD8 T cells on tumor growth was determined as described in the Methods section. Data show the change in tumor volume (mean + SE) of 5 mice/group. *, p < 0.05; **, p <0.01 compared with the control group.

Two experiments were performed to explore the functional activity of these CD8 T cells. Splenocytes from mice in each group were isolated and stimulated *ex vivo* with the CT26-derived AH-1 tumor peptide. To monitor CTL activity, the frequency of IFNγ secreting cells was determined by ELIspot assay. Consistent with changes in the frequency of CD8 T cells noted above, the number of cells stimulated by AH-1 peptide to produce IFNγ was >8-fold higher in mice treated with CpG ODN plus 3M-052 than in controls by 3 days post treatment (p < 0.001, Figure 3). To evaluate the relevance of these T cells *in vivo,* mice that had been challenged with tumor and treated with the combination CpG ODN plus 3M-052 were injected with anti-CD8 Abs. As seen in Figure 4, protection was abrogated by depletion of CD8^+^ but not CD4^+^ T cells, indicating that tumor-specific CD8 T cells were critical mediators of tumor immunity.

### Effect of TLR agonists on gene expression in the tumor microenvironment

Treatment with CpG ODN and/or 3M-052 led to a significant changes in the frequency of CD8 T cells, NK cells and MDSC (Figure 2). To evaluate the activity of these cells, the expression of genes associated with their immunological function was examined by qPCR. The genes selected to evaluate CD8 and NK cell responses were IL-12 and IFNγ (which contribute to the induction and maintenance of immunity) and granzyme B (which mediates their cytotoxicity) [24-26]. As seen in Table 1, cells isolated from the tumor of mice treated with either CpG ODN or 3M-052 had higher levels of expression of IL-12, IFNγ and granzyme B than tumor infiltrating cells from untreated mice (p <.05). In animals treated with a combination of both agonists, mRNA levels were significantly higher when compared to either agonist alone (see Table legend). This effect was additive for IL-12 and IFNγ and supra-additive for Granzyme B.

**Table 1:** Effect of TLR agonists on gene regulation in the tumor microenvironment

	**Mean fold-change in mRNA levels (vs PBS treated controls)**		
**Gene**	**CpG ODN +** **3M control**	**3M-052 + CpG control**	**CpG ODN +** **3M-052**
IL-12	2.23* ± 0.18	1.40 ± 0.19	3.39*** ± 0.11
INFγ	1.94* ± 0.15	1.87* ± 0.25	2.81** ± 0.24
Granzyme B	1.69* ± 0.07	1.87* ± 0.08	4.44*** ± 0.23
Arg1	1.01 ± 0.09	0.63* ± 0.09	0.43** ± 0.08
Nos2	1.83 ± 0.05	0.39** ± 0.04	0.09** ± 0.04
CTLA-4	0.85* ± 0.03	0.52** ± 0.01	0.33*** ± 0.02
TGFβ	0.43* ± 0.03	0.45* ± 0.01	0.26** ± 0.02

Mice were treated as described in Figure 1. mRNA was isolated from tumor infiltrating cells one day after the second treatment and analyzed by RT-PCR. Each point represents the mean ± SD fold difference in cells from treated vs untreated tumor bearing mice derived from independently studying 6 mice/group in 2 independent experiments.

*, p < 0.05; **, p <0.01, ***, p <0.001 when compared to PBS treated controls. Note: the level of expression of all genes from mice treated with CpG ODN plus 3M-052 was also significantly different (p < .01 - 0.05) from that of mice treated with CpG ODN alone or 3M-052 alone.

It is well established that the mechanism by which MDSC suppress T cell cytotoxicity in the tumor microenvironment is mediated by the production of L-arginine via arginase-1 and the release of iNOS [27,28]. The expression of Arg1 and Nos2 by tumor infiltrating immune cells was therefore evaluated by qPCR. Results show that 3M-052 but not CpG ODN reduced the level of expression of genes encoding these immunosuppressive agents (Table 1). The combination of CpG ODN plus 3M-052 further reduced expression levels of both genes, an effect culminating in a nearly 90% reduction in Nos2 mRNA (p <.05).

Immune suppression in the tumor microenvironment can take many forms. One metric of the down-regulation of CTL activity is the expression of CTLA-4 by T cells and another is the production of the immunoinhibitory molecule TGFβ. CTLA-4, a homologue and antagonist of CD28 [29,30], acts as negative regulator of T cell activation by depriving them of CD28-mediated co-stimulation [30,31]. On the other hand, TGFβ suppresses both innate and adaptive immune responses in the tumor microenvironment. CTL-mediated tumor elimination is thus reduced by the presence of TGFβ [32,33]. As both 3M-052 and CpG ODN tend to reduce the level of immune suppression in the tumor microenvironment, their effect on CTLA-4 and TGFβ expression was examined. When compared to cells isolated from tumors treated with PBS, both TLR agonists mediated a significant reduction in the level of expression of these genes (Table 1). The combination of both 3M-052 and CpG ODN was even more effective (p < .05).

### Effect of TLR agonists on the growth of large tumors

**Figure 5:**
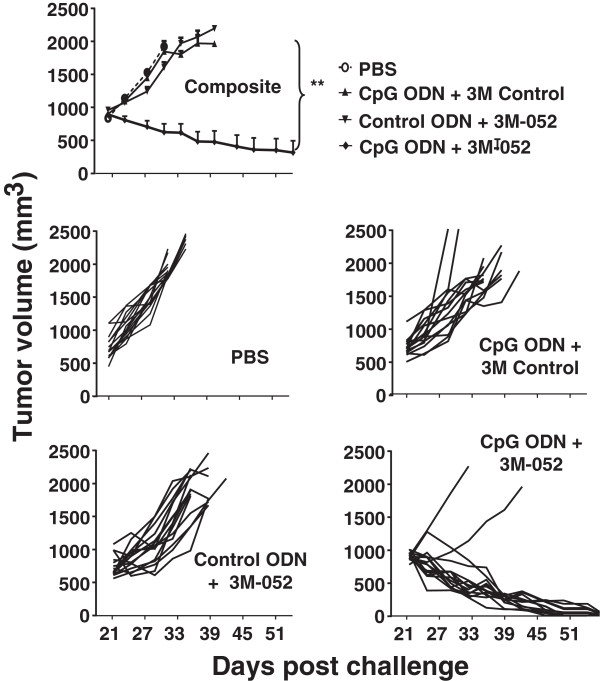
**Effect of TLR agonists on large established CT26 tumors.** 10^5^ CT26 colon cancer cells were implanted into the flank of syngeneic BALB/c mice. When the tumors reached ≈ 800 mm^3^ in volume they were injected with 200 μg of CpG or control ODN and/or 100 μg of 3M-052 or 3M control twice weekly for one month. The change in tumor volume of 15 mice/group is shown (mean + SE).

To evaluate the effect of TLR agonists on tumors of clinically relevant size, CT26 cancer cells were implanted as described above and treatment initiated only after the resultant tumors reached ≈ 800 mm^3^ in volume. Mice were then injected intra-tumorally twice weekly for one month with 200 μg of CpG ODN and/or 100 μg of 3M-052. Tumors in untreated mice proliferated rapidly over this period, reaching a volume of >2,000 mm^3^ within 10 days (mandating their sacrifice as per ACUC guidelines, Figure 5). While both CpG ODN and 3M-052 therapy slowed tumor growth and prolonged survival, tumors in all animals reached 2,000 mm^3^ by 3 wk after the initiation of treatment (Figure 5). In contrast, 87% (13/15) of the mice treated with the combination of CpG ODN plus 3M-052 in 3 independent experiments completely rejected their tumors (p < 0.01; Figure 5).

**Figure 6:**
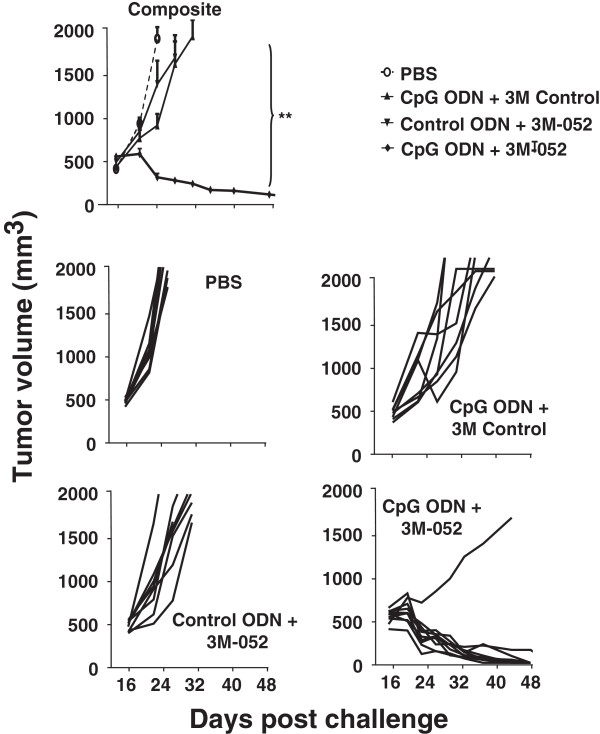
**Effect of TLR agonists on large established B16-F10 tumors.** 10^5^ B16-F10 melanoma cancer cells were implanted into the flank of syngeneic C57Bl/6 mice. When the tumors reached ≈ 500 mm^3^ in volume they were injected with 200 μg of CpG or control ODN and/or 100 μg of 3M-052 or 3M control twice weekly for one month. The change in tumor volume of 8 mice/group is shown (mean + SE).

To verify the utility of this combination against even more aggressive tumors, the studies were repeated in C57/BL6 mice challenged with B16-F10 tumor cells. Therapy was initiated when these tumors reached ≈ 500 mm^3^ in volume. These cancers grow so rapidly that they all reached the 2,000 mm^3^ endpoint in control mice and had to be sacrificed in less than one wk (Figure 6). The same endpoint was reached by all animals treated with a single TLR agonist within 2 wk. In contrast, nearly 90% recipients (8/9) of the combination therapy survived indefinitely, totally clearing their tumors (Figure 6 and Additional file 5: Figure S4).

**Figure 7:**
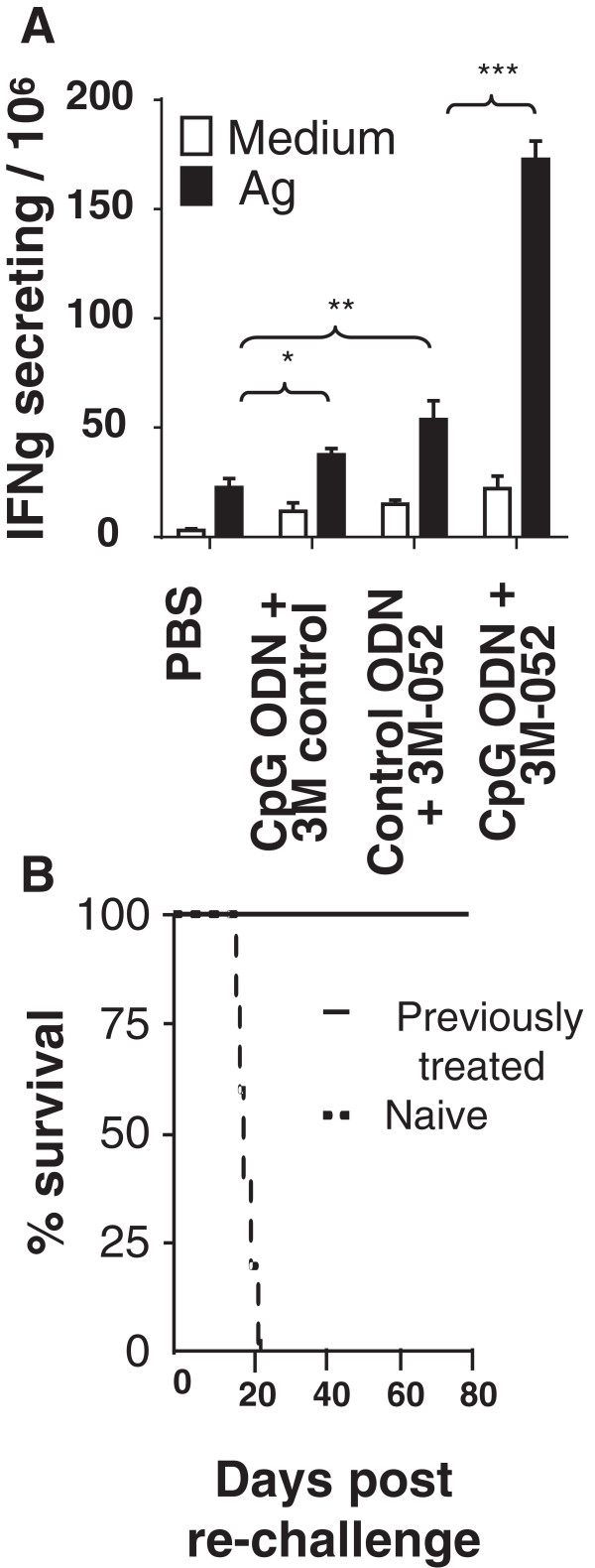
**TLR agonist therapy induces persistent immunity.** 10^5^ CT26 cells were implanted into the flank of syngeneic BALB/c mice. Large established tumors (≈800 mm^3^ in volume) were treated as described in Figure 5. **A)** Cells from the tumor draining LN were isolated one day after the third treatment, stimulated *ex vivo* with AH-1 peptide, and monitored for IFNγ secretion by ELIspot assay. Results represent the mean + SD of 4 independently studied mice/group. **B)** Mice cured of their CT26 tumors by treatment with CpG ODN plus 3M-052 (a cure being defined as being free of detectable tumor for ≥2 months after the cessation of therapy) were re-challenged with 10^6^ CT26 cells. Their survival compared to naive mice challenged with the same tumor dose is shown (N = 6 mice/group). *, p < 0.05; **, p <0.01, ***, p <0.001.

Two approaches were taken to verify that TLR-induced tumor-specific immunity was responsible for these cures. First, lymphocytes were isolated from the draining LN of mice challenged with CT26 tumors one week after the initiation of therapy. These cells were then stimulated *in vitro* with AH-1 peptide and their production of IFNγ monitored. As in Figure 3, T cells from mice treated with the combination of CpG ODN plus 3M-052 generated significantly stronger tumor specific responses that did any of the controls (p <.001, Figure 7A).The twice weekly combination therapy with CpG ODN plus 3M-052 was discontinued when tumors could no longer be detected (generally after 1 month). There was no recurrence of these cancers through 3 months of follow up. To verify that these mice had developed long lasting tumor-specific immunity, they were re-challenged with a 10-fold higher dose of CT26 cells. As seen in Figure 7B, all of these animals survived whereas naive controls perished.

## Discussion

Individual TLR agonists can significantly improve the host’s response to small tumors [34-36]. In the hope of identifying a pairing of agonists that might be effective against large established tumors, a number of TLR agonist combinations were examined. Based on preliminary studies, the combination of 3M-052 plus CpG ODN was selected for further analysis. The anti-tumor activity of CpG ODN includes i) the stimulation of pDC that improve the generation of tumoricidal NK and CD8 T cells and ii) triggering MDSC to differentiate into M1 macrophages that no longer mediate immune suppression [19,20,22,37,38]. TLR 7/8 agonists also support the induction of cancer-specific immunity by triggering an innate response characterized by the production of Th1 cytokines (including TNFα, IL-12, and IFNγ) and DC maturation [39]. Of interest, TLRs 7, 8 and 9 are expressed on different subsets of immune cells that together include T cells, DCs, NK and NKT cells, all of which contribute to anti-tumor activity [40,41].

Whereas many forms of immunotherapy are effective against small tumors (<300 mm^3^), activity wanes when larger tumors are targeted. A number of factors contribute to the resistance of established tumors. In addition to challenging the immune system with a larger number of target cells, organized tumors are better able to cloak themselves in immunosuppressive Tregs and MDSC [42,43]. In this context, MDSC from patients with advanced tumors are particularly effective at inhibiting tumor-specific CD8 T cells [44]. Having found that intra-tumoral delivery of CpG ODN was considerably more effective than systemic administration for the treatment of tumors, our plan was to examine whether adding a TLR 7/8 agonist could further improve this therapeutic approach. Unfortunately, first generation TLR 7/8 agonists were water soluble and proved ineffective when co-administered with CpG ODN. A relatively new TLR 7/8 agonist was identified that contains a modified tail allowing it to persist *in vivo* after being injected into the tumor (3M-052) [23]. Further studies therefore evaluated the activity of locally administered 3M-052 in combination with CpG ODN.The value of combination therapy was initially examined under conditions where a single TLR agonist only delayed tumor growth (Figure 1). Large tumors were then studied in which the combination of CpG ODN plus 3M-052 proved highly successful against both CT26 colon cancer and B10-F16 melanomas. Whereas each agonist alone barely delayed the progression of these large tumors, cure rates on the order of 80 - 90% were achieved by combination therapy. Indeed, as weeping of the injected material from the tumor site was sometimes observed, it is possible that even higher success rates might be achieved by technical improvements in TLR agonist delivery. Successful therapy of large tumors required twice-weekly treatment with CpG ODN plus 3M-052 over the course of ≈ 1 month. A single dose had no detectable effect on the growth of large tumors while 1–2 wks of combination therapy resulted in only short-lived tumor regression. Systemic treatment was uniformly unsuccessful.

There are several mechanisms by which TLR agonists can support the elimination of established tumors. CD28 is a co-stimulatory molecule that enhances the proliferation, cytokine production and survival of TCR-activated T cells. This process is antagonized by CTLA-4, a surface receptor that is up-regulated when T cells become activated [30,31]. We observed that the level of mRNA encoding CTLA-4 was significantly reduced in mice receiving combination therapy (Table 1). This down-modulation of CTLA-4 may help explain the improved activity of tumor-specific T cells found in the current work (Figure 3), consistent with previous findings [45]. We also observed a decrease in mRNA encoding the immunosuppressive cytokine TGFβ in mice treated with CpG ODN plus 3M-052. TGFβ is produced by tumor cells and Gr-1+ CD11b+ MDSC in the tumor microenvironment and serves to suppress both innate and adaptive arms of the immune system [29,46,47]. Consistent with current findings, reduced TGFβ signaling is known to enhance tumor elimination by improving CTL activity [32,33].

To establish the role of increased CTL function in recipients of combination therapy, cells from the tumor draining lymph node were isolated and stimulated *ex vivo* with the CT26-specific AH-1 peptide. While CpG ODN and 3M-052 alone boosted the number of cells secreting IFNγ, significantly more cells from recipients of combination therapy were stimulated to produce that cytokine (Figures 3 and 7A). Consistent with the conclusion that these cells contribute to tumor eradication, the level of mRNA encoding cytokines that promote Th1 and cellular immunity (IL-12 and IFNγ) and the lytic activity of NK and CD8 T cells (granzyme B) were all significantly up-regulated in the tumor microenvironment (Table 1) as were the number of tumor infiltrating CD8 T cells and NK cells (Figure 2).

Despite the above findings, the mechanism(s) by which engagement of TLRs 7, 8 and 9 synergistically enhance anti-tumor immunity will require further investigation. Since all three TLRs utilize the MyD88 dependent signaling pathway, it might seem unlikely that cells expressing receptors for all three agonists could be responsible for such synergy (as any single TLR agonist would be sufficient to trigger such cells). Yet recent studies of TLR expression by individual pDC indicates that phenotypically identical cells nevertheless express very different levels of each receptor, and that engagement of multiple receptors may be necessary to reach a critical activation threshold. Moreover, certain cells express TLR 7 or 8 but not TLR 9 (such as iNKT cells) while MDSC express high levels of TLR 9 but only low levels of TLR 7/8 [22,48].

Other examples of TLR synergy have been observed. For example, co-delivery of the TLR3 agonist poly A:U induced an effective anti-tumor response when used in combination with CpG ODN under conditions when each agonist alone was ineffective [49]. It was also shown that combinations of TLR 2, 3 and 9 ligands could enhance DC function and the induction of T cell immunity following vaccination [50]. Finally, a DC based vaccine delivered with TLR3 and TLR 2 provided enhanced protection to mice challenged with tumor [51].

A number of clinical trials have explored the activity of CpG ODN in cancer patients. Results indicate that CpG treatment induces a dose-related increase in serum levels of IP-10, IFNa, MIP-1a, and IL-12p40 [52,53]. While anti-tumor activity was observed in several phase II trials [54] this finding was not reproduced in a definitive phase III study [55,56]. Of note, none of these studies combined CpG ODN with a TLR7/8 agonist and generally administered the ODN systemically rather intra-tumorally. We postulate that the local delivery of combination TLR 7/8/9 agonists is critical for improving the host’s anti-tumor response by acting on multiple cell types in the tumor microenvironment, including mMDSC, CD8 T lymphocytes and NK cells. Of interest, MDSC express receptors for both agonists and play a vital role protecting tumors from immune aggression by inhibiting T and NK cell activity [22,57]. Current findings demonstrate that the combination of CpG ODN plus 3M-052 reduced mMDSC frequency by 10-fold when compared to untreated mice and 3–5 fold when compared to either agonist alone (Figure 2). This reduction was associated with a significant decline iNOS and arginase-1 expression (Table 1), a constellation of findings that may explain the increase in tumor-specific CTL activity in mice treated with combination therapy (Figures 3 and 7A).

## Conclusions

This work shows that co-administering CpG ODN with 3M-052 is remarkably effective at eliminating large established tumors. This anti-tumor activity is associated with a significant diminution in the frequency of tumor resident MDSC and accumulation of tumor-lytic NK and CD8 T cells, resulting in persistent anti-tumor immunity. These findings indicate that combination TLR immunotherapy may be of considerable benefit in cases of advanced cancer.

## Methods

### Reagents

3M-052 was supplied by 3M Drug Delivery Systems Division as a 4 mg/ml stock solution in ethanol. Endotoxin-free phosphorothioate ODN were synthesized at the Core Facility of the Center for Biologics Evaluation and Research, Food and Drug Administration (Bethesda, MD). The sequences used were: CpG ODN 1555 (5′-GCTAGACGTTAGCGT-3′) and control ODN 1612 (5′-GCTAGAGCTTAGCGT-3′). All ODN were dissolved in PBS at a final concentration of 4 mg/ml.

### Mice and tumor cell lines

6–8 wk old BALB/c and C57BL/6 mice were obtained from the National Cancer Institute (Frederick, MD). The CT26 colon cancer cell line was a kind gift from Dr. Zack Howard (National Cancer Institute) and B16-F10 cell line was purchased from American Type Culture collection (Manassas, VA). Tumor cell lines were maintained in RPMI 1640 medium supplemented with 10% FCS, 100 U/ml penicillin, 100 µg/ml streptomycin, 25 mM HEPES, 1.0 mM sodium pyruvate, nonessential amino acids, and 0.0035% 2-ME. All studies were approved by the National Cancer Institute Frederick Animal Care and Use Committee.

### Tumor experiments

Balb/c mice were injected with 10^5^ CT26 tumor cells while C57BL/6 mice received 10^5^ B16-F10 tumor cells. All injections were s.c. into the right flank. Treatment was initiated when tumors reached a defined size (usually after 2–3 wk). Tumor size was calculated by the formula: (length × width × depth)/2 and mice whose tumor exceeded a diameter of 2.0 cm were euthanized as per ACUC regulations. Two treatment regimens were used. For small tumors (<300 mm^3^), two doses of 100 μg of CpG ODN (4 mg/ml) and/or 50 μg of 3M-052 (4 mg/ml) were injected intra-tumorally using a 30 g needle. To deplete CD4^+^ or CD8^+^ T cell subsets, mice were injected i.p. with 25 ul ascites of rat anti-mouse CD4 (L3/T4) or mouse anti-mouse CD8 (Ly2.2) Abs from Cedarlane labs (Burlington, NC) on day -2, 0, 3 and 6 post-tumor implantation. For large tumors (500–800 mm^3^), 200 μg of CpG ODN and/or 100 μg of 3M-052 were injected intra-tumorally twice weekly for one month. Inactive controls for each TLR agonist were included in all experiments. Tumor growth curves were generated from five mice per group and all results were derived by combining data from 2–3 independent experiments.

### Flow cytometric analysis

Leukocytic infiltrates of the tumor site were prepared by surgical removal of tumor tissue followed by homogenization using a GentleMACS Dissociator (Miltenyi Biotec) and then digestion in RPMI containing 5% fetal calf serum, 250 U/mL type IV collagenase (Invitrogen) and 100 mg/mL DNase I (Roche Molecular Biochemicals) at 37°C for 30 minutes. The resulting single cell suspension was passed through a 70 µm cell strainer (BD Biosciences, Bedford, MA), and washed twice with RPMI. Live cells were isolated by density gradient centrifugation (Histopaque-1077, Sigma-Aldrich), washed, and stained using the following Abs from BD Pharmingen (clone names provided in parentheses). CD11b (M1/70) and Gr-1 (RB6-8C5) for MDSC, CD3 (145-2C11) and CD8 (53–6.7) for T cells and CD49b (DX5) for NK cells. CD45 (30-F11) was used as a leukocyte marker. Stained cells were analyzed using an LSR-II flow cytometer (Becton Dickinson).

### ELISpot assay

Single cell suspensions were prepared from whole spleen, tumor-infiltrating leukocytes or tumor draining lymph nodes and 1.5 - 3.0 × 10^5^ cells/well stimulated for 12 hr with the class-I restricted CT26-derived AH-1 peptide (1 ug/ml) in 96 well Immulon II plates (Millipore, Billerica, MA) coated with anti-IFN Ab (R4-6A2) (BD Biosciences). The plates were washed and treated with biotinylated polyclonal goat anti-IFNγ Ab (R & D systems, MN) followed by streptavidin alkaline phosphatase. Spots were visualized by the addition of a 5-bromo-4-chloro-3-indolyl phosphatase solution (Sigma Aldrich) in low melt agarose (Sigma Aldrich) and counted manually under ×40 magnification. The number of cytokine secreting cells was determined by a single blind reader, and all data was generated by analyzing 12 separate wells per sample.

### Quantitative real-time PCR analysis

Total RNA was isolated from tumor infiltrating cells one day after the second treatment using TRIzol reagent (Invitrogen), precipitated, and then reverse transcribed with Reverse Transcription Kit (Qiagen). IL-12p40, IFNγ, Granzyme B, Arg1, Nos2, CTLA4 and TGFb mRNA levels were examined using the TaqMan Gene Expression Master Mix and the StepOne RT-PCR system (Applied Biosystems, Foster City, CA). All primer sets were from Applied Biosystems. Gene expression was normalized to the level of the GAPDH housekeeping gene. Data were analyzed by StepOne software (Applied Biosystems) and expressed as a fold change in mRNA expression relative to control values. Ct values for all genes studied fell in the range of 22–35.

### Statistical analysis

P values for each experimental group were determined by comparison to the PBS control group using an unpaired student’s t test.

## Abbreviations

CTL: Cytotoxic T cell; DC: Dendritic cell; MDSC: Myeloid derived suppressor cell; ODN: Oligonucleotide; PAMP: Pathogen-associated molecular pattern; TLR: Toll-like receptor.

## Competing interests

Dr. Klinman and members of his lab are co-inventors on a number of patents concerning CpG ODN and their use. All rights to these patents have been assigned to the Federal government. Drs. Smirnov and Vasilakos are employed by 3M Corporation, which holds patents on TLR 7/8 agonists.

## Authors’ contributions

GZ conducted the mouse studies and helped draft the manuscript. JF provided essential reagents and helped design the experiments. DV and JPV established the activity of 3M-052. DMK conceived of and designed the experiments, interpreted the results and helped draft the manuscript. All authors read and approved the final manuscript.

## Authors’ information

Submitting author: John P. Vasilakos.

## Supplementary Material

Additional file 1: Figure S1 In vivo persistence of 3M-052. Serum levels of 3M-052 and Resiquimod were measured at multiple time points after subcutaneous administration of 1 mg/Kg of each agent. Blood was collected before and at various time post delivery. % maximal serum concentration was calculated by the formula: serum level/maximum serum level × 100%. Results represent the mean of 5 independently studied animals/group. Click here for file

Additional file 2: Table S1 HEK293 cells were stably transfected to express human TLR7 or TLR8. Cells were cultured in 24 well plates at 10^6^ cells/mL and treated with 3 µM of 3M-052, Resiquimod, or vehicle (0.33% DMSO). IL-8 levels in culture supernants were measured after 16 hr by ELISA. Results show the mean ± SD of 3 independent cultures. Click here for file

Additional file 3: Figure S2 Gating strategy used to identify the immune cells. Single cell suspensions were prepared as described in the Methods section. Live cells isolated by density gradient centrifugation were stained and analyzed using an LSR-II flow cytometer. The gates used to identify specific cell subpopulations are shown. Click here for file

Additional file 4: Figure S3 TLR agonist therapy does not affect T_reg_ frequency. Mice were treated as described in Figure 1. The frequency of tumor-infiltrating T_reg_ was determined one day after the second treatment by staining for Foxp3^+^ cells. Results show the mean + SD of as a percentage of total CD45^+^ tumor infiltrating cells analyzed independently in 6 mice from 2 independent experiments. Click here for file

Additional file 5: Figure S4 Effect of TLR agonists on large established tumors. Survival curves are provided for mice challenged with CT26 colon cancer cells (A) or B16-F10 melanoma cancer cells (B) and treated with 200 μg of CpG or control ODN and/or 100 μg of 3M-052 or 3M control twice weekly for one month as described in Figures 4 and 5. **; p < .01 vs all 3 control groups. Click here for file

## References

[B1] Janeway CA Jr Medzhitov R Innate immune recognition Annu Rev Immunol 2002 20 197 216 11861602 10.1146/annurev.immunol.20.083001.084359

[B2] Janssens S Beyaert R Role of Toll-like receptors in pathogen recognition Clin Microbiol Rev 2003 16 637 646 14557290 10.1128/CMR.16.4.637-646.2003 PMC207104

[B3] Galluzzi L Vacchelli E Eggermont A Fridman WH Galon J Sautes-Fridman C Tartour E Zitvogel L Kroemer G Trial Watch: Experimental Toll-like receptor agonists for cancer therapy Oncoimmunology 2012 1 699 716 22934262 10.4161/onci.20696 PMC3429574

[B4] Hemmi H Takeuchi O Kawai T Sato S Sanjo H Matsumoto M Hoshino K Wagner H Takeda K Akira S A Toll-like receptor recognizes bacterial DNA Nature 2000 408 740 745 11130078 10.1038/35047123

[B5] Akira S Uematsu S Takeuchi O Pathogen recognition and innate immunity Cell 2006 124 783 801 16497588 10.1016/j.cell.2006.02.015

[B6] Gilliet M Cao W Liu YJ Plasmacytoid dendritic cells: sensing nucleic acids in viral infection and autoimmune diseases Nat Rev Immunol 2008 8 594 606 18641647 10.1038/nri2358

[B7] Smits EL Ponsaerts P Berneman ZN Van TV The use of TLR7 and TLR8 ligands for the enhancement of cancer immunotherapy Oncologist 2008 13 859 875 18701762 10.1634/theoncologist.2008-0097

[B8] Boonstra A sselin-Paturel C Gilliet M Crain C Trinchieri G Liu YJ O’Garra A Flexibility of mouse classical and plasmacytoid-derived dendritic cells in directing T helper type 1 and 2 cell development: dependency on antigen dose and differential toll-like receptor ligation J Exp Med 2003 197 101 109 12515817 10.1084/jem.20021908 PMC2193804

[B9] Matsushima H Yamada N Matsue H Shimada S TLR3-, TLR7-, and TLR9-mediated production of proinflammatory cytokines and chemokines from murine connective tissue type skin-derived mast cells but not from bone marrow-derived mast cells J Immunol 2004 173 531 541 15210814 10.4049/jimmunol.173.1.531

[B10] Gorden KB Gorski KS Gibson SJ Kedl RM Kieper WC Qiu X Tomai MA Alkan SS Vasilakos JP Synthetic TLR agonists reveal functional differences between human TLR7 and TLR8 J Immunol 2005 174 1259 1268 15661881 10.4049/jimmunol.174.3.1259

[B11] Hornung V Rothenfusser S Britsch S Krug A Jahrsdorfer B Giese T Endres S Hartmann G Quantitative expression of toll-like receptor 1–10 mRNA in cellular subsets of human peripheral blood mononuclear cells and sensitivity to CpG oligodeoxynucleotides J Immunol 2002 168 4531 4537 11970999 10.4049/jimmunol.168.9.4531

[B12] Diebold SS Kaisho T Hemmi H Akira S Sousa R e Innate antiviral responses by means of TLR7-mediated recognition of single-stranded RNA Science 2004 303 1529 1531 14976261 10.1126/science.1093616

[B13] Lehner M Morhart P Stilper A Petermann D Weller P Stachel D Holter W Efficient chemokine-dependent migration and primary and secondary IL-12 secretion by human dendritic cells stimulated through Toll-like receptors J Immunother 2007 30 312 322 17414322 10.1097/01.cji.0000211345.11707.46

[B14] Klinman DM Yi A Beaucage SL Conover J Krieg AM CpG motifs present in bacterial DNA rapidly induce lymphocytes to secrete IL-6, IL-12 and IFNg Proc Natl Acad Sci U S A 1996 93 2879 2883 8610135 10.1073/pnas.93.7.2879 PMC39727

[B15] Krieg AM The role of CpG motifs in innate immunity Cur Op Immunol 2000 12 35 43 10.1016/s0952-7915(99)00048-5 10679406

[B16] Maurer T Heit A Hochrein H Ampenberger F O’Keeffe M Bauer S Lipford GB Vabulas RM Wagner H CpG-DNA aided cross-presentation of soluble antigens by dendritic cells Eur J Immunol 2002 32 2356 2364 12209649 10.1002/1521-4141(200208)32:8<2356::AID-IMMU2356>3.0.CO;2-Z

[B17] Baines J Celis E Immune-mediated tumor regression induced by CpG-containing oligodeoxynucleotides Clin Cancer Res 2003 9 2693 2700 12855649

[B18] Lonsdorf AS Kuekrek H Stern BV Boehm BO Lehmann PV Tary-Lehmann M Intratumor CpG-oligodeoxynucleotide injection induces protective antitumor T cell immunity J Immunol 2003 171 3941 3946 14530311 10.4049/jimmunol.171.8.3941

[B19] Heckelsmiller K Rall K Beck S Schlamp A Seiderer J Jahrsdorfer B Krug A Rothenfusser S Endres S Hartmann G Peritumoral CpG DNA elicits a coordinated response of CD8 T cells and innate effectors to cure established tumors in a murine colon carcinoma model J Immunol 2002 169 3892 3899 12244187 10.4049/jimmunol.169.7.3892

[B20] Kawarada Y Ganss R Garbi N Sacher T Arnold B Hammerling GJ NK- and CD8(+) T cell-mediated eradication of established tumors by peritumoral injection of CpG-containing oligodeoxynucleotides J Immunol 2001 167 5247 5253 11673539 10.4049/jimmunol.167.9.5247

[B21] Carpentier A Laigle-Donadey F Zohar S Capelle L Behin A Tibi A Martin-Duverneuil N Sanson M Lacomblez L Taillibert S Puybasset L van Effenterre R Delattre JY Carpentier AF Phase 1 trial of a CpG oligodeoxynucleotide for patients with recurrent glioblastoma Neuro Oncol 2006 8 60 66 16443949 10.1215/S1522851705000475 PMC1871923

[B22] Shirota Y Shirota H Klinman DM Intratumoral injection of CpG oligonucleotides induces the differentiation and reduces the immunosuppressive activity of myeloid-derived suppressor cells J Immunol 2012 188 1592 1599 22231700 10.4049/jimmunol.1101304 PMC3273593

[B23] Smirnov D Schmidt JJ Capecchi JT Wightman PD Vaccine adjuvant activity of 3M-052: an imidazoquinoline designed for local activity without systemic cytokine induction Vaccine 2011 29 5434 5442 21641953 10.1016/j.vaccine.2011.05.061

[B24] Trinchieri G IL-12: a cytokine produced by antigen-presenting cells with inmmunoregulatory functions in the generation of T-helpr cells type 1 and cytotoxic lymphocytes Blood 1994 84 4008 4027 7994020

[B25] Komita H Homma S Saotome H Zeniya M Ohno T Toda G Interferon-gamma produced by interleukin-12-activated tumor infiltrating CD8 + T cells directly induces apoptosis of mouse hepatocellular carcinoma J Hepatol 2006 45 662 672 16935390 10.1016/j.jhep.2006.05.018

[B26] Packard BZ Telford WG Komoriya A Henkart PA Granzyme B activity in target cells detects attack by cytotoxic lymphocytes J Immunol 2007 179 3812 3820 17785818 10.4049/jimmunol.179.6.3812

[B27] Rodriguez PC Ochoa AC Arginine regulation by myeloid derived suppressor cells and tolerance in cancer: mechanisms and therapeutic perspectives Immunol Rev 2008 222 180 191 18364002 10.1111/j.1600-065X.2008.00608.x PMC3546504

[B28] Bronte V Zanovello P Regulation of immune responses by L-arginine metabolism Nat Rev Immunol 2005 5 641 654 16056256 10.1038/nri1668

[B29] Li Z Pang Y Gara SK Achyut BR Heger C Goldsmith PK Lonning S Yang L Gr-1 + CD11b + cells are responsible for tumor promoting effect of TGF-beta in breast cancer progression Int J Cancer 2012 131 2584 2595 22487809 10.1002/ijc.27572 PMC3433574

[B30] Walunas TL Lenschow DJ Bakker CY Linsley PS Freeman GJ Green JM Thompson CB Bluestone JA CTLA-4 can function as a negative regulator of T cell activation Immunity 1994 1 405 413 7882171 10.1016/1074-7613(94)90071-x

[B31] Masteller EL Chuang E Mullen AC Reiner SL Thompson CB Structural analysis of CTLA-4 function in vivo J Immunol 2000 164 5319 5327 10799894 10.4049/jimmunol.164.10.5319

[B32] Gorelik L Flavell RA Immune-mediated eradication of tumors through the blockade of transforming growth factor-beta signaling in T cells Nat Med 2001 7 1118 1122 11590434 10.1038/nm1001-1118

[B33] Nam JS Terabe M Kang MJ Chae H Voong N Yang YA Laurence A Michalowska A Mamura M Lonning S Berzofsky JA Wakefield LM Transforming growth factor beta subverts the immune system into directly promoting tumor growth through interleukin-17 Cancer Res 2008 68 3915 3923 18483277 10.1158/0008-5472.CAN-08-0206 PMC2586596

[B34] Kaczanowska S Joseph AM Davila E TLR agonists: our best frenemy in cancer immunotherapy J Leukoc Biol 2013 93 847 863 23475577 10.1189/jlb.1012501 PMC3656332

[B35] So EY Ouchi T The application of Toll like receptors for cancer therapy Int J Biol Sci 2010 6 675 681 21060729 10.7150/ijbs.6.675 PMC2974170

[B36] Cheng YS Xu F Anticancer function of polyinosinic-polycytidylic acid Cancer Biol Ther 2010 10 1219 1223 20930504 10.4161/cbt.10.12.13450

[B37] Vollmer J Krieg AM Immunotherapeutic applications of CpG oligodeoxynucleotide TLR9 agonists Adv Drug Deliv Rev 2009 61 195 204 19211030 10.1016/j.addr.2008.12.008

[B38] Grauer OM Molling JW Bennink E Toonen LW Sutmuller RP Nierkens S Adema GJ TLR ligands in the local treatment of established intracerebral murine gliomas J Immunol 2008 181 6720 6729 18981089 10.4049/jimmunol.181.10.6720

[B39] Hemmi H Kaisho T Takeuchi O Sato S Sanjo H Hoshino K Horiuchi T Tomizawa H Takeda K Akira S Small anti-viral compounds activate immune cells via the TLR7 MyD88-dependent signaling pathway Nat Immunol 2002 3 196 200 11812998 10.1038/ni758

[B40] Birmachu W Gleason RM Bulbulian BJ Riter CL Vasilakos JP Lipson KE Nikolsky Y Transcriptional networks in plasmacytoid dendritic cells stimulated with synthetic TLR 7 agonists BMC Immunol 2007 8 26 17935622 10.1186/1471-2172-8-26 PMC2175514

[B41] Stary G Bangert C Tauber M Strohal R Kopp T Stingl G Tumoricidal activity of TLR7/8-activated inflammatory dendritic cells J Exp Med 2007 204 1441 1451 17535975 10.1084/jem.20070021 PMC2118597

[B42] Zhou G Drake CG Levitsky HI Amplification of tumor-specific regulatory T cells following therapeutic cancer vaccines Blood 2006 107 628 636 16179369 10.1182/blood-2005-07-2737 PMC1895618

[B43] Lyman MA Aung S Biggs JA Sherman LA A spontaneously arising pancreatic tumor does not promote the differentiation of naive CD8+ T lymphocytes into effector CTL J Immunol 2004 172 6558 6567 15153470 10.4049/jimmunol.172.11.6558

[B44] Almand B Clark JI Nikitina E van Beynen J English NR Knight SC Carbone DP Gabrilovich DI Increased production of immature myeloid cells in cancer patients: a mechanism of immunosuppression in cancer J Immunol 2001 166 678 689 11123353 10.4049/jimmunol.166.1.678

[B45] Rudd CE Taylor A Schneider H CD28 and CTLA-4 coreceptor expression and signal transduction Immunol Rev 2009 229 12 26 19426212 10.1111/j.1600-065X.2009.00770.x PMC4186963

[B46] Bierie B Moses HL Tumour microenvironment: TGFbeta: the molecular Jekyll and Hyde of cancer Nat Rev Cancer 2006 6 506 520 16794634 10.1038/nrc1926

[B47] Flavell RA Sanjabi S Wrzesinski SH Licona-Limon P The polarization of immune cells in the tumour environment by TGFbeta Nat Rev Immunol 2010 10 554 567 20616810 10.1038/nri2808 PMC3885992

[B48] Grela F Aumeunier A Bardel E Van LP Bourgeois E Vanoirbeek J Leite-de-Moraes M Schneider E Dy M Herbelin A Thieblemont N The TLR7 agonist R848 alleviates allergic inflammation by targeting invariant NKT cells to produce IFN-gamma J Immunol 2011 186 284 290 21131420 10.4049/jimmunol.1001348

[B49] Conforti R Ma Y Morel Y Paturel C Terme M Viaud S Ryffel B Ferrantini M Uppaluri R Schreiber R Combadiere C Chaput N Andre F Kroemer G Zitvogel L Opposing effects of toll-like receptor (TLR3) signaling in tumors can be therapeutically uncoupled to optimize the anticancer efficacy of TLR3 ligands Cancer Res 2010 70 490 500 20068181 10.1158/0008-5472.CAN-09-1890

[B50] Zhu Q Egelston C Vivekanandhan A Uematsu S Akira S Klinman DM Belyakov IM Berzofsky JA Toll-like receptor ligands synergize through distinct dendritic cell pathways to induce T cell responses: implications for vaccines Proc Natl Acad Sci U S A 2008 105 16260 16265 18845682 10.1073/pnas.0805325105 PMC2570973

[B51] Lim SN Kuhn S Hyde E Ronchese F Combined TLR stimulation with Pam3Cys and Poly I: C enhances Flt3-ligand dendritic cell activation for tumor immunotherapy J Immunother 2012 35 670 679 23090076 10.1097/CJI.0b013e318270e135

[B52] Krieg AM Antitumor applications of stimulating toll-like receptor 9 with CpG oligodeoxynucleotides Curr Oncol Rep 2004 6 88 95 14751085 10.1007/s11912-004-0019-0

[B53] Offersen R Melchjorsen J Paludan SR Ostergaard L Tolstrup M Sogaard OS TLR9-adjuvanted pneumococcal conjugate vaccine induces antibody-independent memory responses in HIV-infected adults Hum Vaccin Immunother 2012 8 1042 1047 22854665 10.4161/hv.20707 PMC3551874

[B54] Krieg AM Efler SM Wittpoth M Al Adhami MJ Davis HL Induction of systemic TH1-like innate immunity in normal volunteers following subcutaneous but not intravenous administration of CPG 7909, a synthetic B-class CpG oligodeoxynucleotide TLR9 agonist J Immunother 2004 27 460 471 15534490 10.1097/00002371-200411000-00006

[B55] Hirsh V Paz-Ares L Boyer M Rosell R Middleton G Eberhardt WE Szczesna A Reiterer P Saleh M Arrieta O Bajetta E Webb RT Raats J Benner RJ Fowst C Meech SJ Readett D Schiller JH Randomized phase III trial of paclitaxel/carboplatin with or without PF-3512676 (Toll-like receptor 9 agonist) as first-line treatment for advanced non-small-cell lung cancer J Clin Oncol 2011 29 2667 2674 21632509 10.1200/JCO.2010.32.8971

[B56] Manegold C van Zandwijk N Szczesna A Zatloukal P Au JS Blasinska-Morawiec M Serwatowski P Krzakowski M Jassem J Tan EH Benner RJ Ingrosso A Meech SJ Readett D Thatcher N A phase III randomized study of gemcitabine and cisplatin with or without PF-3512676 (TLR9 agonist) as first-line treatment of advanced non-small-cell lung cancer Ann Oncol 2012 23 72 77 21464154 10.1093/annonc/mdr030

[B57] Li H Han Y Guo Q Zhang M Cao X Cancer-expanded myeloid-derived suppressor cells induce anergy of NK cells through membrane-bound TGF-beta J Immunol 2009 182 240 249 19109155 10.4049/jimmunol.182.1.240

