# Supplementary figures and images for: Combination therapy targeting toll like receptors 7, 8 and 9 eliminates large established tumors
Source: J Immunother Cancer. 2014 May 13;2:12. doi: 10.1186/2051-1426-2-12 (PMC4075973; doi:10.1186/2051-1426-2-12)

Supplemental Figure 1

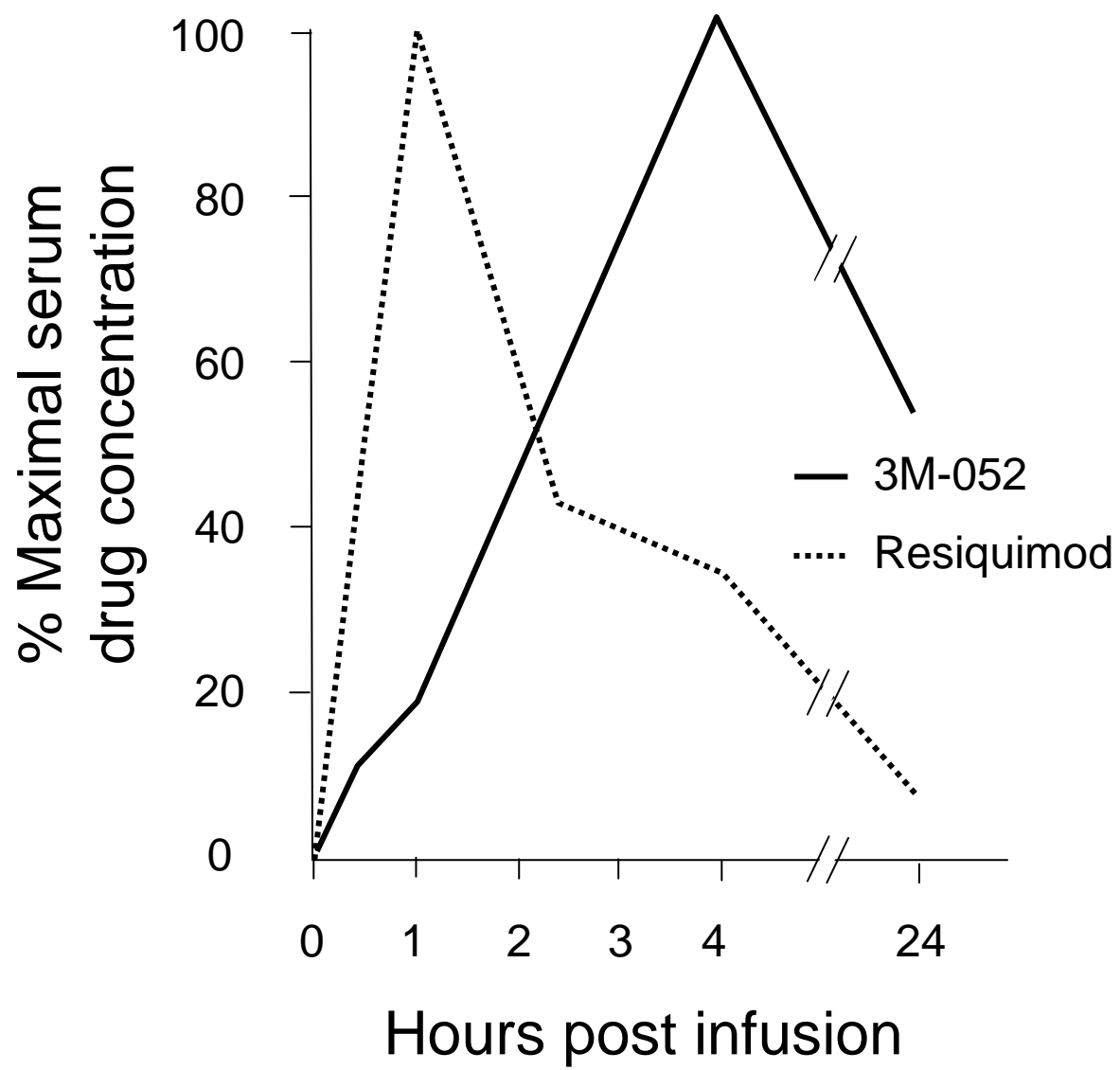

Supplement: Additional file 1: Figure S1 — In vivo persistence of 3M-052. Serum levels of 3M-052 and Resiquimod were measured at multiple time points after subcutaneous administration of 1 mg/Kg of each agent. Blood was collected before and at various time post delivery. % maximal serum concentration was calculated by the formula: serum level/maximum serum level × 100%. Results represent the mean of 5 independently studied animals/group. [file 2051-1426-2-12-S1.pdf]

Supplemental Figure 2

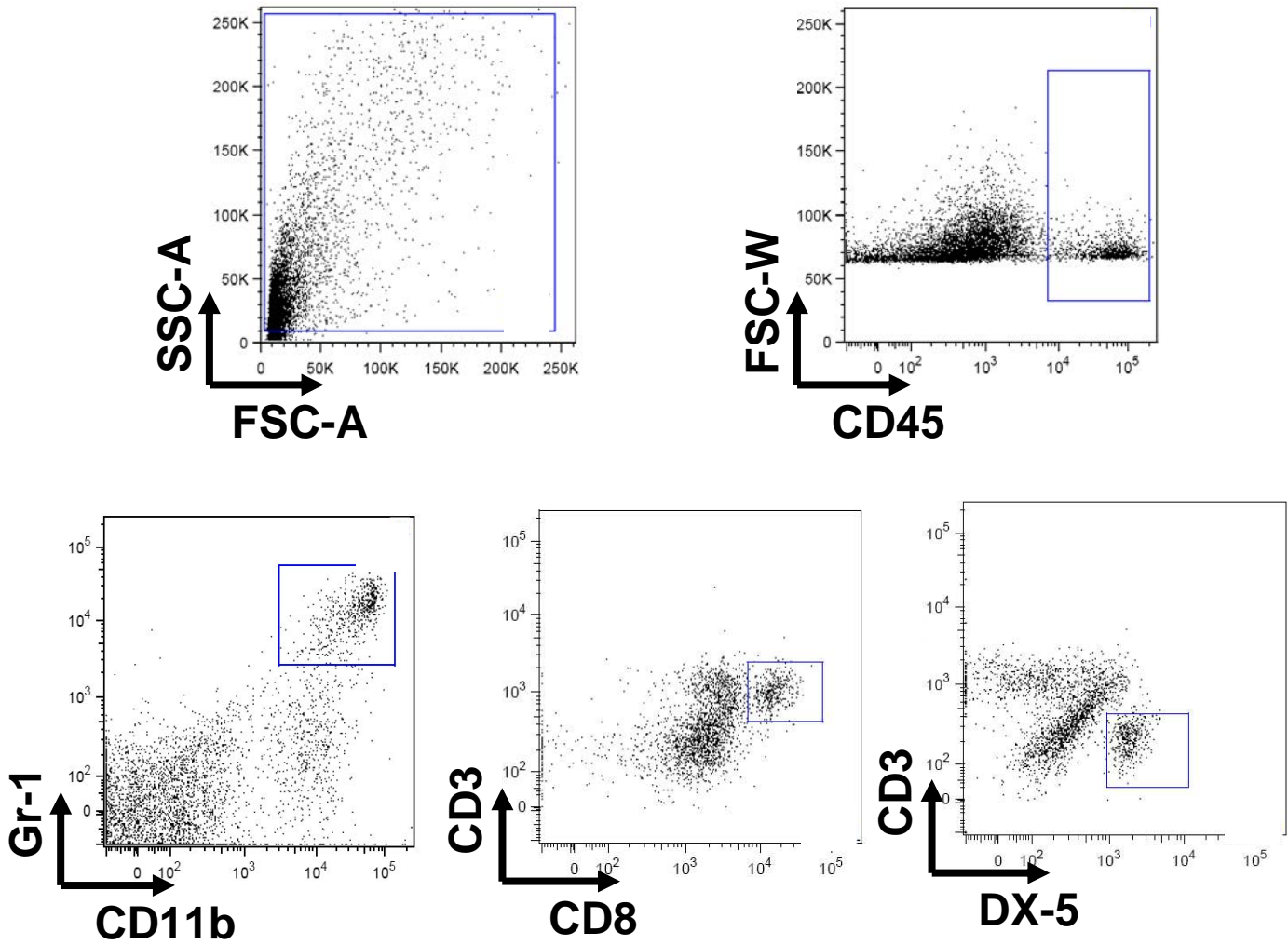

Supplement: Additional file 3: Figure S2 — Gating strategy used to identify the immune cells. Single cell suspensions were prepared as described in the Methods section. Live cells isolated by density gradient centrifugation were stained and analyzed using an LSR-II flow cytometer. The gates used to identify specific cell subpopulations are shown. [file 2051-1426-2-12-S3.pdf]

Supplemental Figure 3

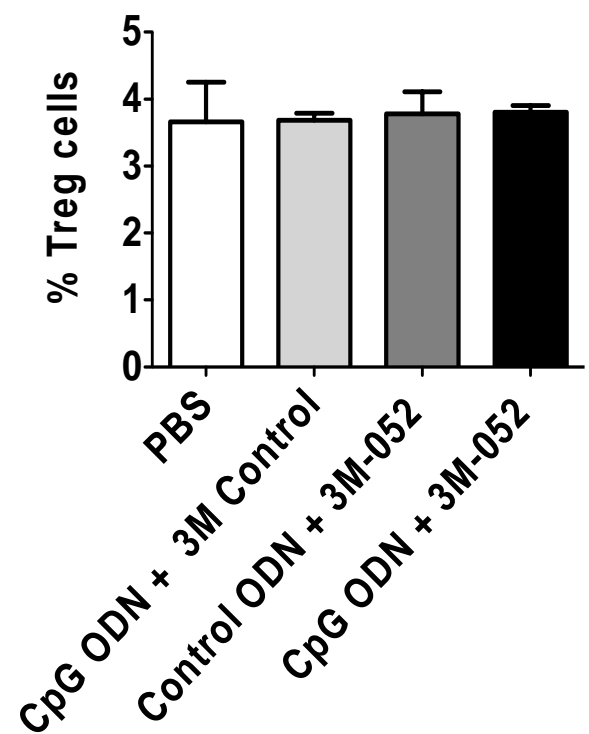

Supplement: Additional file 4: Figure S3 — TLR agonist therapy does not affect Treg frequency. Mice were treated as described in Figure 1. The frequency of tumor-infiltrating Treg was determined one day after the second treatment by staining for Foxp3+ cells. Results show the mean + SD of as a percentage of total CD45+ tumor infiltrating cells analyzed independently in 6 mice from 2 independent experiments. [file 2051-1426-2-12-S4.pdf]

Supplemental Figure 4

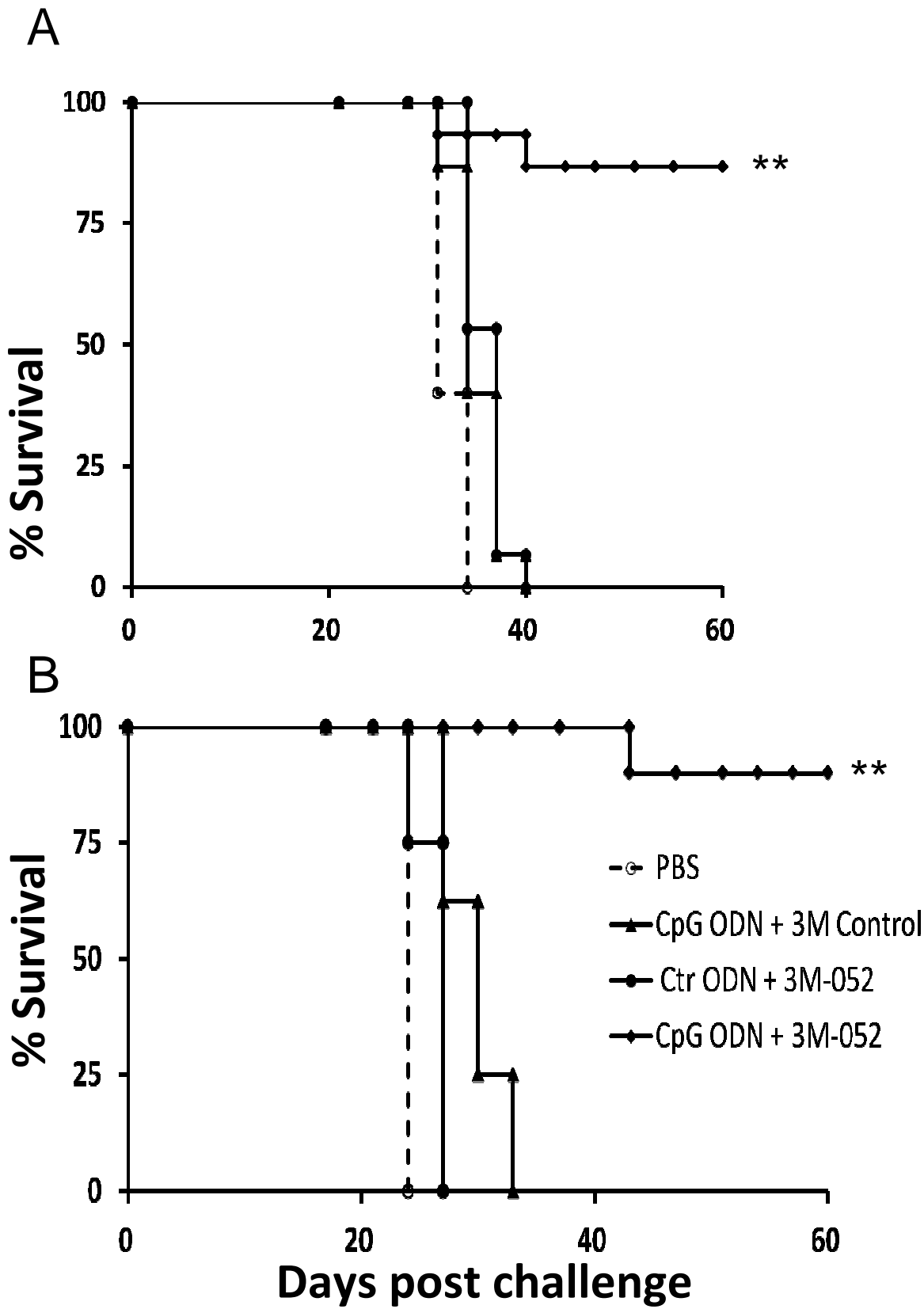

Supplement: Additional file 5: Figure S4 — Effect of TLR agonists on large established tumors. Survival curves are provided for mice challenged with CT26 colon cancer cells (A) or B16-F10 melanoma cancer cells (B) and treated with 200 μg of CpG or control ODN and/or 100 μg of 3M-052 or 3M control twice weekly for one month as described in Figures 4 and 5. **; p < .01 vs all 3 control groups. [file 2051-1426-2-12-S5.pdf]
