# Supplementary material for: Combination therapy targeting toll like receptors 7, 8 and 9 eliminates large established tumors
Source: J Immunother Cancer. 2014 May 13;2:12. doi: 10.1186/2051-1426-2-12 (PMC4075973; doi:10.1186/2051-1426-2-12)
Supplement: Additional file 2: Table S1 — HEK293 cells were stably transfected to express human TLR7 or TLR8. Cells were cultured in 24 well plates at 106 cells/mL and treated with 3 µM of 3M-052, Resiquimod, or vehicle (0.33% DMSO). IL-8 levels in culture supernants were measured after 16 hr by ELISA. Results show the mean ± SD of 3 independent cultures. [file 2051-1426-2-12-S2.doc]

Table S1

IL-8 production (pg/ml)

Sample Untransfected TLR7 expressing TLR8 expressing

HEK cells HEK cells HEK cells

Vehicle 99 + 13 109 + 20 99 + 7

3M-052 81 + 19 887 + 269 2374 + 691

Resiquimod 90 + 4 2157 + 716 3193 + 1135

HEK293 cells were stably transfected to express human TLR7 or TLR8. Cells were cultured in 24 well plates at 10^6^ cells/mL and treated with 3 uM of 3M-052, Resiquimod, or vehicle (0.33% DMSO). IL-8 levels in culture supernants were measured after 16 hr by ELISA. Results show the mean + SD of 3 independent cultures.
